# CYP2D6 phenotype and post-surgical pain control with hydrocodone and oxycodone

**DOI:** 10.3389/fphar.2026.1817652

**Published:** 2026-05-11

**Authors:** Christelle Lteif, Rachel A. Myers, Erica N. Elwood, Elizabeth C. Harris, Hrishikesh Chakraborty, Paul R. Dexter, Josh F. Peterson, Renee Rider, Todd C. Skaar, Simona Volpi, Julie A. Johnson, Julio D. Duarte, Larisa H. Cavallari

**Affiliations:** 1 Department of Pharmacotherapy and Translational Research and Center for Pharmacogenomics and Precision Medicine, College of Pharmacy, University of Florida, Gainesville, FL, United States; 2 Duke Clinical Research Institute, Duke University School of Medicine, Durham, NC, United States; 3 School of Medicine, Indiana University, Indianapolis, IN, United States; 4 Departments of Biomedical Informatics and Medicine, Vanderbilt University Medical Center, Nashville, TN, United States; 5 Division of Genomic Medicine, National Human Genome Research Institute, Bethesda, MD, United States; 6 Division of Clinical Pharmacology, Indiana University School of Medicine, Indianapolis, IN, United States; 7 Clinical and Translational Science Institute, The Ohio State University, Columbus, OH, United States; 8 Department of Internal Medicine, The Ohio State University College of Medicine, Columbus, OH, United States; 9 Department of Pharmaceutics and Pharmacology, The Ohio State University College of Pharmacy, Columbus, OH, United States

**Keywords:** CYP2D6, hydrocodone, oxycodone, pain control, post-surgery

## Abstract

**Background:**

Hydrocodone and oxycodone are widely used for acute postoperative pain and are metabolized by CYP2D6 to more potent agonists. The clinical impact of CYP2D6 metabolizer status on hydrocodone and oxycodone analgesic response remains uncertain. This secondary analysis of A Depression and Opioid Pragmatic Trial in Pharmacogenetics (ADOPT PGx) evaluated associations between CYP2D6 phenotype and postoperative pain and opioid consumption among patients taking hydrocodone or oxycodone.

**Methods:**

This analysis included participants from the ADOPT PGx Acute Pain Trial (NCT05966129). Among patients who consumed hydrocodone or oxycodone postoperatively, analyses were conducted separately by drug, comparing outcomes between poor (PM) vs. normal (NM) and intermediate (IM) vs. normal CYP2D6 metabolizers, with phenotypes predicted by genotype and concomitant CYP2D6 inhibitor use. A positive control analysis was performed among tramadol users. Outcomes were cumulative opioid use (morphine milligram equivalents) and composite Patient-Reported Outcomes Measurement Information System® pain intensity scores at 10 days post-surgery. Regression models adjusted for demographics, surgery type, trial site, and non-opioid analgesic use; *P*-values were corrected for multiple comparisons.

**Results:**

The cohort primarily underwent orthopedic procedures and concomitant non-opioid analgesic use was common across hydrocodone, oxycodone, and tramadol cohorts, with 88%–100% of patients receiving at least one non-opioid analgesic or nerve block. Among hydrocodone users, predicted CYP2D6 phenotype was not associated with postoperative hydrocodone consumption (PM vs. NM: mean ratio [MR] = 1.12, 95% CI 0.92–1.36, *P* = 0.262) or pain intensity (PM vs. NM: odds ratio [OR] = 1.13, 95% CI 0.66–1.92, *P* = 0.668). Among oxycodone users, CYP2D6 phenotype was not associated with postoperative oxycodone consumption (PM vs. NM: MR = 1.02, 95% CI 0.79–1.34, *P* = 0.880) or pain intensity (PM vs. NM: OR = 0.84, 95% CI 0.47–1.51, *P* = 0.568). Similarly, in the positive control tramadol cohort, CYP2D6 phenotype was not associated with tramadol consumption (PM vs. NM: MR = 1.02, 95% CI 0.79–1.32, *P* = 0.847) or pain intensity (PM vs. NM: OR = 1.43, 95% CI 0.66–3.00, *P* = 0.668).

**Conclusion:**

Predicted CYP2D6 metabolizer status was not associated with postoperative pain control or opioid use in patients taking hydrocodone, oxycodone, or positive-control tramadol, suggesting that multimodal analgesic use may have weakened pharmacogenetic effects. These findings suggest that CYP2D6 phenotype associations with hydrocodone or oxycodone analgesic response, if present, are attenuated in multimodal postoperative pain management.

## Introduction

Hydrocodone and oxycodone are among the most commonly prescribed opioids for the management of acute post-surgical pain (Torabi et al., 2019). Both agents undergo metabolism via the CYP2D6 enzyme to more potent agonists of the mu-opioid receptor, with hydrocodone primarily O-demethylated to hydromorphone and oxycodone metabolized to oxymorphone (Crews et al., 2021). *CYP2D6*, which encodes CYP2D6, is highly polymorphic, contributing to observed interindividual variability in enzyme activity (Crews et al., 2021). Genotype-predicted CYP2D6 intermediate (IMs) or poor (PMs) metabolizers exhibit reduced or absent enzyme function, respectively, which limits conversion of hydrocodone and oxycodone to their more active metabolites and may potentially impair analgesic response (Coates and Lazarus, 2023). Although pharmacokinetic data show that reduced CYP2D6 activity is associated with lower concentrations of opioid active metabolites, the clinical impact of CYP2D6 phenotype on hydrocodone- and oxycodone-related pain outcomes remains uncertain (Crews et al., 2021; Balyan et al., 2017; Zhu et al., 2022). Therefore, current Clinical Pharmacogenetics Implementation Consortium (CPIC) guidelines do not provide recommendations for oxycodone therapy based on CYP2D6 phenotype, and while hydrocodone is addressed, the available evidence supports only an optional recommendation to consider alternative analgesia in IMs or PMs not responding well to initial hydrocodone treatment (Crews et al., 2021).

A Depression and Opioid Pragmatic Trial in Pharmacogenetics (ADOPT PGx) Acute Pain Trial (NCT05966129), a large pragmatic randomized controlled trial conducted by the Implementing GeNomics In pracTicE (IGNITE) Network, evaluated CYP2D6 phenotype-guided prescribing of opioids in CYP2D6 IMs/PMs and found no significant improvement in post-surgical pain outcomes with a guided approach compared with usual care (control) (Cavallari et al., 2022; Cavallari et al., 2026). The present study aimed to evaluate the association between CYP2D6 phenotype (based on genotype and drug interactions) and post-surgical pain control and opioid consumption among individuals taking hydrocodone or oxycodone in the ADOPT PGx Acute Pain Trial.

## Methods

### CYP2D6 associations with hydrocodone and oxycodone

These secondary analyses were conducted using data from the ADOPT PGx Acute Pain Trial, a prospective, multicenter, pragmatic, open-label randomized clinical trial of CYP2D6-guided versus usual prescribing for post-surgical pain control, as previously described (Cavallari et al., 2022; Cavallari et al., 2026). Briefly, the ADOPT PGx Acute Pain Trial was conducted between March 2021 and September 2023 at eight U.S. health systems and compared CYP2D6 phenotype-guided prescribing versus usual care (control) for postoperative pain, with a composite measure of opioid consumption and pain control as the primary outcome (Cavallari et al., 2022; Cavallari et al., 2026). Patients undergoing surgical procedures with an expected duration of postoperative pain across the 10-day follow-up period were prioritized for enrollment (Cavallari et al., 2022; Cavallari et al., 2026). The protocol was approved by the Duke University Health System Institutional Review Board (IRB), which served as the IRB of record for all participating sites, and all participants provided written informed consent (Cavallari et al., 2022; Cavallari et al., 2026). The current analyses focused on participants who were prescribed and reported taking hydrocodone or oxycodone, with outcomes evaluated according to phenotype-predicted CYP2D6 metabolizer status. Participants were included if primary outcome data were available at the 10-day post-operative time point.

*CYP2D6* genotype in this study was derived from a panel of common functionally relevant alleles (*2, *3, *4, *5, *6, *10, *17, *41) and copy number variation, capturing the majority of clinically relevant functional variation in *CYP2D6* (Cavallari et al., 2022). The CYP2D6 activity score (AS) was assigned using CPIC star allele activity values, with scores summed across alleles to generate the genotype-predicted enzyme activity (Relling and Klein, 2011; Relling et al., 2020). Normal metabolizers (NMs; historically referred to as extensive metabolizers) were defined as having a CYP2D6 AS of 1–2, inclusive (Cavallari et al., 2022). Intermediate metabolizers (IMs) were defined as having a CYP2D6 AS of 0.25–0.75, inclusive (Cavallari et al., 2022). Poor metabolizers (PMs) were defined as having a CYP2D6 AS of 0 (Cavallari et al., 2022). Comparisons were performed between PMs and NMs as well as between IMs and NMs. To account for differing definitions of CYP2D6 IMs between the ADOPT PGx trial and current CPIC guidelines, additional analyses were conducted using different AS ranges (Torabi et al., 2019): AS > 0-< 1.25 vs. AS 1.25–2.25 (CPIC definitions of IMs vs. NMs), and (Crews et al., 2021) AS > 0–0.75 vs. AS > 0.75-< 1.25 (IMs with lower vs. higher activity scores).

CYP2D6 activity was evaluated using both phenotype-based and genotype-based approaches, with phenotype-based classifications prespecified as the primary analysis. For phenotype-based analyses, AS values were further adjusted for concomitant use of moderate or strong CYP2D6 inhibitors (i.e., phenoconversion), yielding a CYP2D6 phenotype that reflected both genotype and inhibitor effects. Concomitant CYP2D6 inhibitor use was based on medications reported at the10-day postoperative timepoint, reflecting exposure during the postoperative opioid use period. Strong inhibitors considered were bupropion, fluoxetine, paroxetine, quinidine, and terbinafine, while moderate inhibitors included abiraterone, duloxetine, cinacalcet, lorcaserin, and mirabegron, based on the U.S. Food and Drug Administration Table of Inhibitors (U.S. Food and Drug Administration FDA, 2023). In contrast, genotype-based analyses used the genotype-predicted AS and excluded all participants taking moderate or strong CYP2D6 inhibitors, ensuring that these analyses reflected genotype effects alone.

As a positive control, parallel analyses were performed among participants who were prescribed and reported taking tramadol, given the well-documented association between CYP2D6 phenotype and tramadol’s analgesic effectiveness (Crews et al., 2021).

### Pain control outcomes

The primary outcomes of interest were opioid consumption and composite pain intensity. Opioid use was determined by subtracting the number of tablets remaining from the number prescribed at 10 (±3) days after surgery based on patient report; the resulting amount was then converted to morphine milligram equivalents (MME) using hydrocodone-, oxycodone and tramadol-specific conversion factors according to the drug analyzed (Cavallari et al., 2022). Composite pain intensity was assessed via the Patient-Reported Outcomes Measurement Information System (PROMIS®) Pain Intensity score at 10 (±3) days after surgery, incorporating current pain as well as worst and average pain over the past 7 days on a five-point Likert scale (HealthMeasures, 2021).

### Statistical analysis

Associations between CYP2D6 phenotype and outcomes were evaluated using regression models, with covariates selected *a priori* based on potential confounding of pain outcomes. For oxycodone, models were adjusted for age; race; sex; surgical procedure type; trial site; use of acetaminophen, nonsteroidal anti-inflammatory drugs (NSAIDs), and gabapentinoids; concomitant use of tramadol; and nerve block administration. The same model was used for hydrocodone, except that acetaminophen was excluded because all patients received a hydrocodone-acetaminophen combination product. Analyses for tramadol used the same covariates, with the addition of concomitant opioid use, and tramadol omitted as a covariate since it was the drug of interest in this subgroup. In tramadol-specific association analyses, the primary models included all tramadol users, whether or not they were concurrently taking another opioid. Sensitivity analyses were conducted among tramadol-only users, excluding participants taking any other opioids.

For opioid use (MMEs), generalized linear models with a gamma distribution and log link were used because of the positive, right-skewed distribution of MME values. Results were expressed as mean ratios (MRs) with 95% confidence intervals (CIs), representing the relative difference in mean MME across CYP2D6 phenotype groups. For composite pain scores, ordinal logistic regression models were applied, as pain scores were ordered outcomes where the numerical difference between adjacent values did not necessarily reflect an equal difference in pain intensity. Results were presented as odds ratios (ORs) and 95% CIs, representing the odds of reporting a higher pain category. For all comparisons, the CYP2D6 NM phenotype was used as the reference group, with PM and IM phenotypes compared against NM. For analyses using alternative AS ranges, the higher AS group (AS 1.25–2.25 or AS >0.75-<1.25, respectively) was used as the reference. The proportional odds assumption for the ordinal logistic regression was assessed using alternative cumulative link models and found to be reasonable, with results consistent across approaches. Collinearity among predictors was also evaluated using variance inflation factors, and no evidence of problematic collinearity was observed.

To account for multiple comparisons, Bonferroni correction was applied separately within each drug-specific analysis family, defined as all CYP2D6 phenotype comparisons for a given outcome (PM vs. NM, IM vs. NM, and the two AS subgroup contrasts; total of four comparisons per outcome). Statistical significance was defined as *P* < 0.013, corresponding to the adjusted α-level of 0.05/4 = 0.013. To maintain data privacy and adhere to small cell reporting conventions in tables, counts fewer than five were reported as ‘<5’. All analyses were conducted in R version 4.4.1.

## Results

### Study population

Across the hydrocodone, oxycodone, and tramadol cohorts, participants were predominantly postoperative orthopedic patients, with knee arthroplasty representing the most common procedure at 39%–61% across opioid cohorts and CYP2D6 phenotype groups (Tables 1, 2; Supplementary Table S1). Concomitant use of non-opioid analgesics was common, with acetaminophen use ranging from 50.0%–100%, NSAID use from 33%–56%, gabapentinoid use from 17%–44%, and nerve block use, including perioperative and post-discharge nerve blocks, ranging from 5%–58% of patients depending on opioid cohort and CYP2D6 phenotype group (Tables 1, 2; Supplementary Table S1). Overall, 100% of hydrocodone users, 88% of oxycodone users, and 91% of tramadol users received at least one non-opioid analgesic or nerve block.

**TABLE 1:** Clinical characteristics and medication use in surgery patients taking hydrocodone, stratified by CYP2D6 metabolizer status.

Patient characteristic	NM (N = 447)	IM (N = 47)	PM (N = 59)	*P*-value IM vs. NM	*P*-value PM vs. NM
Age (median [IQR])	66.00 [57.50, 72.00]	67.00 [59.00, 73.00]	64.00 [53.50, 71.00]	0.562	0.153
Male (%)	198 (44)	20 (43)	20 (34)	0.941	0.169
Race (%)	​	​	​	0.169	0.879
Black or african american	48 (11)	9 (19)	6 (10)	​	​
White or european american	369 (83)	37 (79)	48 (81)	​	​
Other	30 (7)	<5	5 (9)	​	​
Surgery type (%)	​	​	​	0.56	0.059
Joint replacement hip	155 (35)	13 (28)	13 (22)	​	​
Joint replacement knee	227 (51)	27 (57)	30 (51)	​	​
Other*	65 (15)	7 (15)	16 (27)	​	​
Site (%)	​	​	​	0.551	0.004
Site 1	42 (9)	<5	<5	​	​
Site 2	36 (8)	<5	6 (10)	​	​
Site 3	7 (2)	0 (0)	5 (9)	​	​
Site 4	<5	0 (0)	0 (0)	​	​
Site 5	<5	<5	<5	​	​
Site 6	41 (9)	5 (11)	10 (17)	​	​
Site 7	319 (71)	34 (72)	35 (59)	​	​
NSAIDs (%)	239 (56)	19 (42)	32 (56)	0.084	1
Gabapentinoids (%)	152 (36)	20 (44)	23 (40)	0.365	0.57
Tramadol (%)	34 (8)	<5	<5	0.777	0.603
Nerve block use (%)	​	​	​	0.689	0.961
Never	201 (45)	20 (43)	27 (46)		
During surgery	117 (26)	15 (32)	16 (27)		
After discharge	129 (29)	12 (26)	16 (27)		
Moderate CYP2D6 inhibitors	16 (4)	14 (30)	<5	NA	NA
Strong CYP2D6 inhibitors	0 (0)	0 (0)	35 (59)	NA	NA

* Other/rare surgeries include fracture repair, joint replacement other, mastectomy, osteotomy, and surgeries originally listed as “Other”. Percentages are rounded to whole numbers; totals may not equal 100% due to rounding. IM, CYP2D6 intermediate metabolizer; NA, not applicable; NM, CYP2D6 normal metabolizer; NSAIDs, nonsteroidal anti-inflammatory drugs; PM, CYP2D6 poor metabolizer. *P*-values compare IM, vs. NM, and PM, vs. NM., Normality was assessed using the Shapiro-Wilk test. For continuous variables, t-tests were used unless data were non-normally distributed, in which case non-parametric tests (Wilcoxon/Mann-Whitney) were applied. Chi-square tests were used for categorical variables unless any expected cell count was <5, in which case Fisher’s exact test was applied.

**TABLE 2:** Clinical characteristics and medication use in surgery patients taking oxycodone, stratified by CYP2D6 metabolizer status.

Patient characteristic	NM (N = 249)	IM (N = 44)	PM (N = 53)	*P*-value IM vs. NM	*P*-value PM vs. NM
Age (median [IQR])	61.00 [51.00, 70.00]	66.00 [58.25, 71.00]	61.00 [53.00, 68.00]	0.141	0.6
Male (%)	88 (35)	14 (32)	17 (32)	0.779	0.768
Race (%)	​	​	​	0.095	0.229
Black or african american	40 (16)	13 (30)	4 (8)	​	​
White or european american	169 (68)	27 (61)	42 (79)	​	​
Other	40 (16)	4 (9)	7 (13)	​	​
Surgery type (%)	​	​	​	0.553	0.199
Joint replacement hip	46 (19)	11 (25)	15 (28)	​	​
Joint replacement knee	96 (39)	21 (48)	26 (49)	​	​
Other*	107 (43)	12 (27)	12 (23)	​	​
Site (%)	​	​	​	0.27	0.001
Site 1	24 (10)	5 (11)	8 (15)	​	​
Site 2	26 (10)	7 (16)	11 (21)	​	​
Site 3	5 (2)	0 (0)	0 (0)	​	​
Site 4	80 (32)	9 (21)	5 (9)	​	​
Site 5	8 (3)	0 (0)	0 (0)	​	​
Site 6	56 (23)	16 (36)	22 (42)	​	​
Site 7	50 (20)	7 (16)	7 (13)	​	​
Acetaminophen (%)	146 (59)	26 (59)	34 (64)	1	0.556
NSAIDs (%)	82 (35)	16 (36)	17 (33)	0.943	1
Gabapentinoids (%)	41 (17)	12 (27)	14 (28)	0.175	0.136
Tramadol (%)	49 (21)	11 (25)	11 (22)	0.648	1
Nerve block use (%)	​	​	​	0.185	0.07
Never	113 (45)	19 (43)	17 (32)		
During surgery	111 (45)	24 (55)	33 (62)		
After discharge	25 (10)	<5	<5		
Moderate CYP2D6 inhibitors	<5	10 (23)	<5	NA	NA
Strong CYP2D6 inhibitors	0 (0)	0 (0)	33 (62)	NA	NA

* Other/rare surgeries include fracture repair, joint replacement other, mastectomy, osteotomy, spinal surgery and surgeries originally listed as “Other”. Percentages are rounded to whole numbers; totals may not equal 100% due to rounding. IM, CYP2D6 intermediate metabolizer; NA, not applicable; NM, CYP2D6 normal metabolizer; NSAIDs, nonsteroidal anti-inflammatory drugs; PM, CYP2D6 poor metabolizer. *P*-values compare IM, vs. NM, and PM, vs. NM., Normality was assessed using the Shapiro-Wilk test. For continuous variables, t-tests were used unless data were non-normally distributed, in which case non-parametric tests (Wilcoxon/Mann-Whitney) were applied. Chi-square tests were used for categorical variables unless any expected cell count was <5, in which case Fisher’s exact test was applied.

### Hydrocodone cohort

**FIGURE 1:**
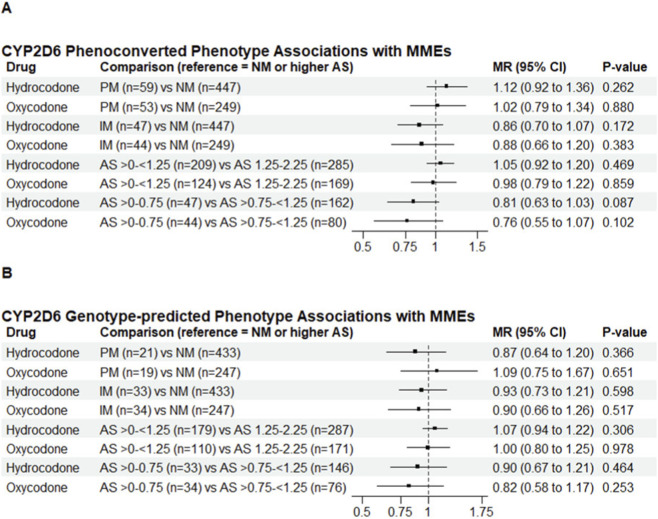
CYP2D6 Phenoconverted and Genotype-predicted Phenotype Associations with Opioid Morphine Milligram Equivalents. Forest plots depict adjusted mean ratios (with 95% confidence intervals) for cumulative hydrocodone or oxycodone morphine milligram equivalents at 10 (±3) days after surgery across CYP2D6 phenotype groups **(A)** Results based on phenoconverted (incorporating inhibitor effect) CYP2D6 phenotypes **(B)** Results based on genotype-predicted phenotypes (excluding patients on CYP2D6 inhibitors). NMs were used as the reference group for phenotype comparisons (PM vs. NM, IM vs. NM). For AS subgroup comparisons, the higher AS group (AS 1.25–2.25 or AS >0.75-<1.25) was used as the reference. MR values <1 indicate lower opioid use relative to the reference group. MRs and CIs were estimated from generalized linear regression models using a gamma distribution, adjusting for age, race, sex, surgical procedure type, trial site group, and concomitant acetaminophen (except for hydrocodone as all patients received hydrocodone-acetaminophen), nonsteroidal anti-inflammatory drugs, gabapentinoids, serotonin and norepinephrine reuptake inhibitors, tramadol, and nerve block administration. AS, activity score; CI, confidence interval; IM, intermediate metabolizer; MMEs, morphine milligram equivalents; MR, mean ratio; NM, normal metabolizer; PM, poor metabolizer.

**FIGURE 2:**
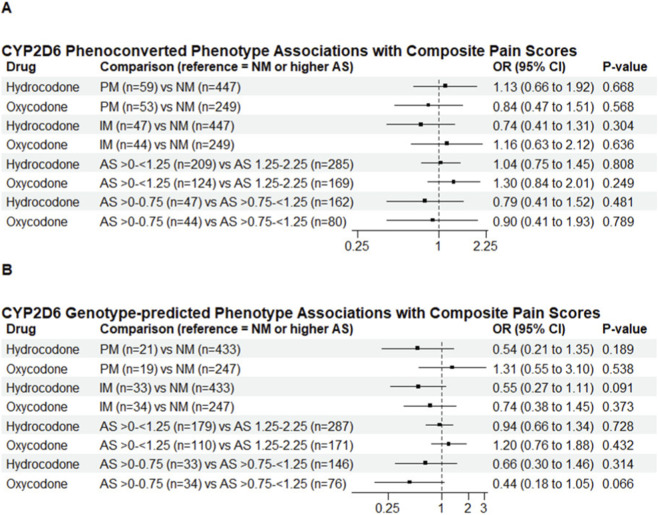
CYP2D6 Phenoconverted and Genotype-predicted Phenotype Associations with Composite Pain Scores. Forest plots depict adjusted odds ratios (with 95% confidence intervals) for composite pain scores at 10 (±3) days after surgery across CYP2D6 phenotype groups for hydrocodone and oxycodone **(A)** Results based on phenoconverted (incorporating CYP2D6 inhibitor effect) CYP2D6 phenotypes **(B)** Results based on genotype-predicted phenotypes (excluding patients on CYP2D6 inhibitors). NMs were used as the reference group for phenotype comparisons (PM vs. NM, IM vs. NM). For AS subgroup comparisons, the higher AS group (AS 1.25–2.25 or AS >0.75-<1.25) was used as the reference. OR values <1 indicate lower pain scores relative to the reference group. ORs and CIs were estimated from ordinal logistic regression models, adjusting for age, race, sex, surgical procedure type, trial site group, and concomitant acetaminophen (except for hydrocodone as all patients received hydrocodone-acetaminophen), nonsteroidal anti-inflammatory drugs, gabapentinoids, serotonin and norepinephrine reuptake inhibitors, tramadol, and nerve block administration. AS, activity score; CI, confidence interval; IM, intermediate metabolizer; NM, normal metabolizer; OR, odds ratio; PM, poor metabolizer.

The hydrocodone cohort included 553 patients (Table 1). Significant differences in the site of enrollment were observed between phenotype-defined PM and NM groups (*P* = 0.004). CYP2D6 phenotype (based on genotype and use of CYP2D6 inhibiting medications) was not significantly associated with postoperative hydrocodone consumption (PM vs. NM: MR = 1.12, 95% CI 0.92–1.36, *P* = 0.262; IM vs. NM: MR = 0.86, 95% CI 0.70–1.07, *P* = 0.172; Figure 1) or pain intensity (PM vs. NM: OR = 1.13, 95% CI 0.66–1.92, *P* = 0.668; IM vs. NM: OR = 0.74, 95% CI 0.41–1.31, *P* = 0.304; Figure 2). Results were similar in genotype-based analyses and across alternative AS range comparisons, with no significant associations observed (Figures 1, 2).

### Oxycodone cohort

The oxycodone cohort included 346 patients (Table 2), with differences in the site of enrollment observed between phenotype-defined PMs and NMs (*P* = 0.001). CYP2D6 phenotype (based on genotype and drug interactions) was not significantly associated with postoperative opioid consumption (PM vs. NM: MR = 1.02, 95% CI 0.79–1.34, *P* = 0.880; IM vs. NM: MR = 0.88, 95% CI 0.66–1.20, *P* = 0.383; Figure 1) or pain intensity (PM vs. NM: OR = 0.84, 95% CI 0.47–1.51, *P* = 0.568; IM vs. NM: OR = 1.16, 95% CI 0.63–2.12, *P* = 0.636; Figure 2). Results were similar in genotype-based analyses and across alternative AS range comparisons, with no significant associations observed (Figures 1, 2).

### Tramadol cohort

The tramadol cohort included 202 patients (Supplementary Table S1). Significant differences in age (*P* = 0.032), race (*P* = 0.041), and enrollment site (*P* = 0.020) were observed between phenotype-defined PM and NM groups, and a significantly higher proportion of patients in the IM group were on gabapentinoids (*P* = 0.026). Dual opioid use was common across phenotype groups. Among CYP2D6 NM tramadol users, 35 (24%) patients were also prescribed hydrocodone and 60 (41%) patients were prescribed oxycodone. Among CYP2D6 IM tramadol users, fewer than 5 (<19%) patients were on hydrocodone, and 15 (58%) patients were on oxycodone; among PMs, fewer than five patients (<18%) were on hydrocodone, and 17 (61%) patients were on oxycodone (Supplementary Table S1).

Among participants receiving tramadol, including those on other opioids, no significant associations were observed between CYP2D6 phenotype (based on genotype and drug interactions) and tramadol consumption (Supplementary Figure S1). A nominal association was observed for pain control, with genotype-predicted IMs having a lower odds of higher postoperative composite pain scores than NMs (OR = 0.36, 95% CI 0.14–0.96, *P* = 0.042; Supplementary Figure S2B); however, this association was not significant in the larger sample of pooled IMs/PMs, and did not remain significant after correction for multiple comparisons.

Sensitivity analyses restricted to those taking tramadol without concomitant use of another opioid showed lower cumulative tramadol MME in IMs vs. NMs (MR = 0.54, 95% CI 0.33–0.91, *P* = 0.019; Supplementary Figure S3A), and similarly lower consumption in AS >0–0.75 vs. AS >0.75-<1.25 (MR = 0.40, 95% CI 0.20–0.83, *P* = 0.016; Supplementary Figure S3A). Genotype-predicted phenotype analyses were limited by small sample sizes of IMs and PMs (N < 5) after excluding patients taking additional opioids; therefore, genotype-only analyses were not conducted. None of the tramadol findings remained statistically significant after correction for multiple comparisons.

## Discussion

This study evaluated the association between CYP2D6 metabolizer phenotype and postoperative opioid consumption and pain intensity among patients who were prescribed and reported taking hydrocodone, oxycodone or tramadol. The study population consisted primarily of patients undergoing orthopedic surgery who were largely managed with multimodal analgesia, commonly including NSAIDs, gabapentinoids, acetaminophen, and/or nerve blocking agents. In this setting of substantial multimodal analgesic use, CYP2D6 metabolizer status was not significantly associated with postoperative opioid consumption or pain control with hydrocodone, oxycodone, or tramadol. The absence of association in the positive control tramadol cohort supports the interpretation that multimodal analgesic strategies may have attenuated or obscured pharmacogenetic effects in acute postoperative pain management.

CYP2D6 converts hydrocodone into hydromorphone and oxycodone into oxymorphone, both of which are more potent analgesic metabolites (Crews et al., 2021; Coates and Lazarus, 2023). CYP2D6 PMs and IMs have reduced hydromorphone and oxymorphone formation (Crews et al., 2021; Balyan et al., 2017; Zhu et al., 2022). However, differences in clinical analgesic response across CYP2D6 genotype-informed metabolizer phenotypes have not resulted in strong clinical recommendations (Crews et al., 2021). CPIC guidelines do not provide recommendations for oxycodone, largely due to inconsistent findings across studies, and classify hydrocodone guidance for IMs and PMs as optional, reflecting the limited number of available studies and their predominantly weak-to-moderate strength at the time of guideline development (Crews et al., 2021). In our acute postoperative cohort, we observed no significant differences in opioid consumption (measured as MME) or pain scores between IMs and NMs, or between PMs and NMs for hydrocodone, oxycodone, or tramadol. To ensure our results were not confounded by differences in IM classification, we also analyzed our data using updated CPIC definitions based on activity score ranges and similarly found no significant differences.

A small number of postoperative studies have similarly reported no significant associations between CYP2D6 phenotype and hydrocodone or oxycodone response post-surgery (Langman et al., 2021; Zwisler et al., 2010; Ali et al., 2023). In a cohort of 336 adult orthopedic surgery and trauma patients prescribed hydrocodone following total knee or total hip replacement or extremity injuries, Langman et al. found no association between CYP2D6 phenotype (genotype-predicted or phenoconverted) and inpatient pain scores or hydrocodone dose requirements. However, the inhibitor-adjusted CYP2D6 IM/PM phenotype was associated with a longer duration of opioid use after discharge compared with CYP2D6 NMs and ultrarapid metabolizers. This latter finding extends beyond the acute postoperative period evaluated in the present study (Langman et al., 2021). Zwisler et al. evaluated 270 adults undergoing primarily thyroid surgery or hysterectomy where CYP2D6 phenotype was determined by genotype, and patients were blinded until study completion (Zwisler et al., 2010). Patients received scheduled acetaminophen and diclofenac in addition to oxycodone, and although PMs generated lower plasma oxymorphone concentrations, their oxycodone consumption, pain relief, and rates of inadequate analgesic response were similar to those of NMs (Zwisler et al., 2010). In a pediatric cohort with orthopedic fractures, *CYP2D6* variation was not associated with differences in pain control among children prescribed oxycodone, as assessed by maximum, minimum, and average post-medication pain scores; however, opioid consumption was not evaluated, and most patients in that cohort received oxycodone combined with acetaminophen (Ali et al., 2023). In contrast, postoperative studies evaluating tramadol have more frequently demonstrated genotype- or phenotype-associated differences in analgesic response (Kamiya et al., 2023; Dong et al., 2015). For example, CYP2D6 IMs exhibited higher postoperative pain scores among 85 Japanese patients following orthopedic surgery (Kamiya et al., 2023), and *CYP2D6* *10/*10 carriers required greater tramadol consumption with higher early postoperative pain scores after nephrectomy in 111 patients (Dong et al., 2015). Overall, existing postoperative studies, including the present analysis, have not identified consistent clinically meaningful associations between CYP2D6 phenotype and hydrocodone or oxycodone response. While *CYP2D6* variation does not appear to significantly influence short-term postoperative outcomes, it remains uncertain whether this reflects a true absence of effect or attenuation by multimodal pain management strategies.

Beyond the postoperative setting, chronic and cancer pain studies provide mixed evidence. In 33 patients with advanced cancer, no differences in oxycodone dose requirements, pain scores, or adverse effects were observed between IM/PMs compared with NMs (Wong et al., 2024). Similarly, in a European cohort of 450 patients with cancer, *CYP2D6* genotype significantly influenced oxymorphone formation, but these pharmacokinetic differences did not translate into differences in analgesia or side effects (Andreassen et al., 2012). In a primary care cohort of adults prescribed codeine, oxycodone, hydrocodone, or tramadol for pain documented in the EHR (pain type and location not specified), CYP2D6 poor and ultrarapid metabolizers were more likely to experience inadequate pain control or adverse reactions, respectively, after adjustment for age and sex (St et al., 2017). Sample sizes limited adjustment for additional variables, but the authors reported that secondary models, including opioid type and total number of prescriptions, yielded similar age- and sex-adjusted associations, although effect estimates were not reported separately to indicate which opioids drove the associations (St et al., 2017). These studies indicate mixed evidence regarding the relationship between CYP2D6-related metabolite formation and clinical outcomes with hydrocodone or oxycodone in patients with cancer pain or non-cancer-related chronic pain.

In contrast, some studies suggest CYP2D6 variation may contribute to differences in opioid effectiveness in broader clinical settings or in studies evaluating multiple opioids. A large population-based analysis of 8,062 hospitalized patients receiving codeine, tramadol, hydrocodone, or oxycodone for various or unclassified pain conditions reported that PMs experienced less pain reduction and required higher opioid doses compared to NMs, but analyses were not stratified by individual opioid (Brady et al., 2003). Similarly, a recent analysis of over 31,000 participants from the All of Us cohort found that reduced CYP2D6 function, defined by phenotype incorporating both genotype and inhibitor exposure, was associated with increased pain-related emergency department visits, primarily in hydrocodone, tramadol, and codeine users, but not in those prescribed oxycodone (Nahid et al., 2025). When examining genotype alone among participants not taking CYP2D6 inhibitors, significant associations were observed for hydrocodone, tramadol, and codeine, with IMs and PMs experiencing more pain-related emergency department visits, but no association was seen for oxycodone. In an analysis where all four opioids were combined, there was no significant effect of CYP2D6 genotype on ED visits. These data suggest CYP2D6 is important for hydrocodone, tramadol and codeine but not oxycodone (Nahid et al., 2025). However, in this same study, co-administration of CYP2D6 inhibitors in NMs was linked to more emergency department visits across all opioids studied, including hydrocodone and oxycodone, suggesting that inhibitor exposure may exert a stronger influence on clinical outcomes than genotype alone in real-world populations (Nahid et al., 2025). The observed effect of CYP2D6 inhibitors is further supported by a recent large electronic health record study, which found that concomitant use of CYP2D6 inhibitors with hydrocodone, tramadol, codeine, or oxycodone was associated with increased pain-related emergency department visits for all four drugs, highlighting the clinical impact of drug-drug interactions across CYP2D6-dependent opioids (Nahid et al., 2024).

Because CYP2D6 inhibitor exposure can phenoconvert patients and has been associated with reduced opioid effectiveness in prior studies, we accounted for inhibitor use in phenotype-predicted CYP2D6 analyses and performed complementary genotype-predicted analyses restricted to participants not taking CYP2D6 inhibitors. Across both approaches, CYP2D6 variation was not associated with postoperative pain outcomes. This finding is consistent with prior oxycodone studies similarly reporting that neither CYP2D6 genotype nor CYP2D6 inhibitor exposure meaningfully affected pain control, despite lower oxymorphone-to-oxycodone ratios in PMs, suggesting that oxymorphone formation may not be essential for oxycodone’s analgesic effect (Smith et al., 2019). When accounting for CYP2D6 inhibitor exposure, no clinically meaningful differences in postoperative analgesic response were observed for both hydrocodone or oxycodone in our cohort. Although larger real-world studies suggest that inhibitor effects may be important across multiple CYP2D6-dependent opioids (Nahid et al., 2025; Nahid et al., 2024), these findings highlight that, in the context of short-term postoperative care with multimodal analgesia, the impact of inhibitors may be attenuated.

Overall, the clinical relevance of *CYP2D6* variation is highly context dependent. In postoperative settings, where non-opioid analgesics were commonly administered alongside opioids in some studies, including the present study, CYP2D6 genotype or phenotype has generally not been associated with significant differences in hydrocodone or oxycodone analgesic response. This contrasts with some findings from chronic pain populations, where longer-term opioid exposure and less reliance on multimodal strategies may allow CYP2D6-related effects to become more apparent, particularly when multiple CYP2D6-metabolized opioids or CYP2D6 inhibitor exposure are considered. These differences in clinical context may explain why pharmacogenetic associations observed outside postoperative care are not consistently reproduced in surgical populations.

In contrast to hydrocodone and oxycodone, the evidence linking tramadol’s analgesic effectiveness to CYP2D6 genotype is more clear, resulting in CPIC guidelines recommending avoidance of tramadol in CYP2D6 PMs because of the high likelihood of an ineffective analgesic response to the drug (Crews et al., 2021). The absence of significant associations between CYP2D6 phenotype and tramadol effectiveness in our study, despite its strong dependence on CYP2D6 metabolism, supports the interpretation that pharmacogenetics effects may be attenuated in postoperative settings where multimodal analgesia is widely used. In this context, effective pain control may be achieved through non-opioid analgesics and nerve blocks, reducing reliance on opioids and potentially limiting the ability to detect CYP2D6 phenotype-dependent differences in opioid response. For example, total knee arthroplasty, the most common procedure in our cohort, is associated with substantial pain; however, multimodal pain management strategies have previously been shown to provide effective analgesia while reducing opioid consumption in this setting (Grosu et al., 2014; Wylde et al., 2018; Lavand’homme et al., 2022) Multimodal analgesia may therefore function as an effect modifier, potentially attenuating CYP2D6-related differences in analgesic response, although formal interaction analyses were not performed due to limited power.

This study has several strengths. It leverages data from the ADOPT PGx trial, a large, prospective, multicenter, pragmatic randomized trial, which enhances the generalizability of our findings to real-world postoperative settings. Analyses were restricted to participants with verified postoperative opioid use, ensuring that including patients who did not report taking any of their prescribed opioid did not dilute potential pharmacogenetic effects. CYP2D6 activity was estimated using both genotype-predicted and phenoconverted phenotypes with multiple activity score definitions, minimizing the risk of misclassification. Statistical models were adjusted for key confounders, including multimodal pain management strategies, and inclusion of hydrocodone, oxycodone, and tramadol cohorts allowed evaluation across opioids with differing CYP2D6 dependence. However, several limitations should be acknowledged. First, these analyses are non-randomized secondary analyses from a pragmatic trial, in which postoperative treatment approaches, including multimodal analgesia, were determined by the prescriber, which may have confounded associations between CYP2D6 phenotype and analgesic outcomes. Sample sizes of PMs and IMs were relatively small in some analyses, particularly among tramadol-only users, limiting statistical power to detect modest associations; however, the observed estimates and confidence intervals suggest that large clinically meaningful effects are unlikely. Opioid consumption was based on patient self-report, which may introduce measurement variability and could limit the ability to detect subtle associations with CYP2D6 phenotype. CYP2D6 phenotype was inferred from genotype and inhibitor exposure rather than direct pharmacokinetic measures, which may result in some misclassification of metabolizer status. In addition, this study evaluated CYP2D6, although opioid response and pain modulation are multifactorial and may also be influenced by other genotypes, such as those for opioid receptors and catechol-O-methyltransferase (Andersen and Skorpen, 2009). Opioid-related genotypes other than *CYP2D6* were not assessed in our cohort, and thus, we were unable to determine their influences on opioid response. However, a comprehensive set of common functionally relevant alleles and copy number variation of *CYP2D6* were assessed in the parent trial, capturing the majority of clinically relevant functional variation. Lastly, the analysis focused on short-term postoperative opioid use in predominantly orthopedic patients, limiting generalizability to other surgical populations who are not prescribed multimodal pain therapy.

In conclusion, in this secondary analysis of the ADOPT PGx Acute Pain trial, CYP2D6 metabolizer phenotype showed no association with post-operative analgesic response to hydrocodone, oxycodone, or tramadol. Overall, these results suggest that CYP2D6-related differences in hydrocodone or oxycodone analgesic response, if present, were not detectable within the context of multimodal postoperative pain management.

## Data Availability

The datasets presented in this study can be found in online repositories. The names of the repository/repositories and accession number(s) can be found below: https://dbgap.ncbi.nlm.nih.gov/home/, phs004058.v1.p1.
