# Supplementary material for: CYP2D6 phenotype and post-surgical pain control with hydrocodone and oxycodone
Source: Front Pharmacol. 2026 May 11;17:1817652. doi: 10.3389/fphar.2026.1817652 (PMC13199760; doi:10.3389/fphar.2026.1817652)
Supplement: Supplementary file 1 [file DataSheet1.pdf]

Supplementary Material

CYP2D6 Phenotype and Post-surgical Pain Control with Hydrocodone and Oxycodone

Christelle Lteif[1], Rachel A. Myers[2], Erica N. Elwood[1], Elizabeth C. Harris[2], Hrishikesh Chakraborty[2], Paul R. Dexter[3], Josh F. Peterson[4], Renee Rider[5], Todd C. Skaar[6], Simona Volpi[5], Julie A. Johnson[1,7,8,9], Julio D. Duarte[1*], Larisa H. Cavallari[1*] for the IGNITE Network Investigators

1. Department of Pharmacotherapy and Translational Research and Center for Pharmacogenomics and Precision Medicine, College of Pharmacy, University of Florida, Gainesville, Florida, USA.
2. Duke Clinical Research Institute, Duke University School of Medicine, Durham, North Carolina, USA.
3. School of Medicine, Indiana University, Indianapolis, Indiana, USA.
4. Departments of Biomedical Informatics and Medicine, Vanderbilt University, Medical Center, Nashville, Tennessee, USA.
5. Division of Genomic Medicine, National Human Genome Research Institute, Bethesda, Maryland, USA.
6. Division of Clinical Pharmacology, Indiana University School of Medicine, Indianapolis, Indiana, USA
7. Clinical and Translational Science Institute, The Ohio State University, Columbus, Ohio, USA.
8. Department of Internal Medicine, The Ohio State University College of Medicine, Columbus, Ohio, USA.
   Department of Pharmaceutics & Pharmacology, The Ohio State University College of Pharmacy, Columbus, Ohio, USA.

***Co-corresponding authors:**

Julio D. Duarte, PharmD, PhD, FAHA
University of Florida College of Pharmacy
HSC PO Box 100486
1600 SW Archer Road, Gainesville, FL 32610
E-mail: juliod@cop.ufl.edu

Larisa H. Cavallari, PharmD, BCPS, FCCP
University of Florida College of Pharmacy
HSC PO Box 100486
1600 SW Archer Road, Gainesville, FL 32610
E-mail: lcavallari@cop.ufl.edu

Supplementary Table 1. Clinical Characteristics and Medication Use in Surgery Patients Taking Tramadol, Stratified by CYP2D6 Metabolizer Status

Patient Characteristic	NM (N=148)	IM (N=26)	PM (N=28)	*P*-value IM vs NM	*P*-value PM vs NM
**Age (median [IQR])**	67.00 [60.00, 74.00]	68.00 [64.25, 71.75]	62.50 [57.00, 67.25]	0.600	0.032
**Male (%)**	55 (37)	9 (35)	7 (25)	0.978	0.308
**Race (%)**				0.801	0.041
Black or African American	15 (10)	4 (15)	0 (0)		
White or European American	126 (85)	21 (81)	24 (86)		
Other	7 (5)	< 5	< 5		
**Surgery type (%)**				0.974	0.548
Joint replacement hip	53 (36)	8 (31)	10 (36)		
Joint replacement knee	66 (45)	14 (54)	17 (61)		
Other*	29 (20)	< 5	< 5		
**Site (%)**				0.458	0.020
Site 1	45 (30)	7 (27)	5 (18)		
Site 2	< 5	< 5	< 5		
Site 3	6 (4)	0 (0)	0 (0)		
Site 4	66 (45)	14 (54)	19 (68)		
Site 5	30 (20)	< 5	< 5		
**Acetaminophen (%)**	99 (67)	13 (50)	18 (64)	0.138	0.923
**NSAIDs (%)**	48 (33)	10 (39)	10 (36)	0.759	0.961
**Gabapentinoids (%)**	29 (20)	11 (42)	6 (21)	0.026	1
**Hydrocodone (%)**	35 (24)	< 5	< 5	0.206	0.330
**Oxycodone (%)**	60 (41)	15 (58)	17 (61)	0.157	0.077
**Nerve Block Use (%)**				0.18	0.528
Never	78 (53)	9 (35)	14 (50)		
During Surgery	62 (42)	15 (58)	14 (50)		
After Discharge	8 (5)	< 5	0 (0)		
**Moderate CYP2D6 Inhibitors**	6 (4)	9 (35)	1 (4)	NA	NA
**Strong CYP2D6 Inhibitors**	0 (0)	0 (0)	22 (79)	NA	NA

*** Other/rare surgeries include joint replacement other, mastectomy, osteotomy, spinal surgery and surgeries originally listed as "Other". Percentages are rounded to whole numbers; totals may not equal 100% due to rounding. IM, CYP2D6 intermediate metabolizer; NA, not applicable; NM, CYP2D6 normal metabolizer; NSAIDs, nonsteroidal anti-inflammatory drugs; PM, CYP2D6 poor metabolizer. *P*-values compare IM vs NM and PM vs NM. Normality was assessed using the Shapiro-Wilk test. For continuous variables, t-tests were used unless data were non-normally distributed, in which case non-parametric tests (Wilcoxon/Mann-Whitney) were applied. Chi-square tests were used for categorical variables unless any expected cell count was <5, in which case Fisher's exact test was applied.**

A

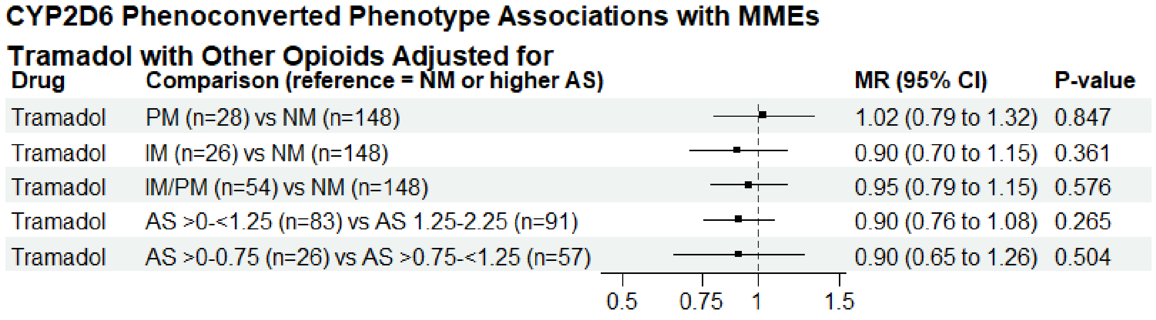

B

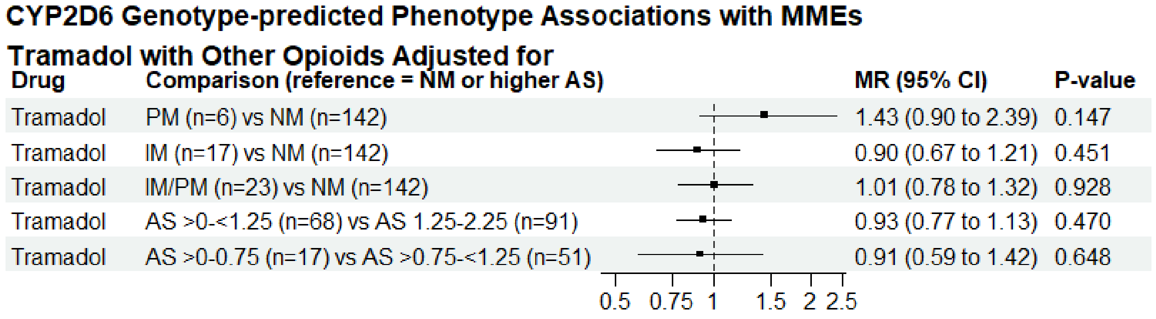

Supplementary Figure 1. CYP2D6 Phenoconverted and Genotype-predicted Phenotype Associations with Tramadol Morphine Milligram Equivalents. Forest plots depict adjusted mean ratios (with 95% confidence intervals) for cumulative tramadol morphine milligram equivalents at 10 (±3) days after surgery, with concomitant use of other opioids included as covariates in all models. (A) Results based on phenoconverted (incorporating CYP2D6 inhibitor effect) CYP2D6 phenotypes; (B) Results based on genotype-predicted phenotypes (excluding patients on CYP2D6 inhibitors). NMs were used as the reference group for phenotype comparisons (PM vs NM, IM vs NM, and IM/PM vs NM). For AS subgroup comparisons, the higher AS group (AS 1.25-2.25 or AS >0.75-<1.25) was used as the reference. MR values <1 indicate lower opioid use relative to the reference group. MRs and CIs were estimated from generalized linear regression models using a gamma distribution, adjusting for age, race, sex, surgical procedure type, trial site group, acetaminophen, nonsteroidal anti-inflammatory drugs, gabapentinoids, serotonin and norepinephrine reuptake inhibitors, nerve block administration, and other opioid use. AS, activity score; CI, confidence interval; IM, intermediate metabolizer; MMEs, morphine milligram equivalents; MR, mean ratio; NM, normal metabolizer; PM, poor metabolizer.

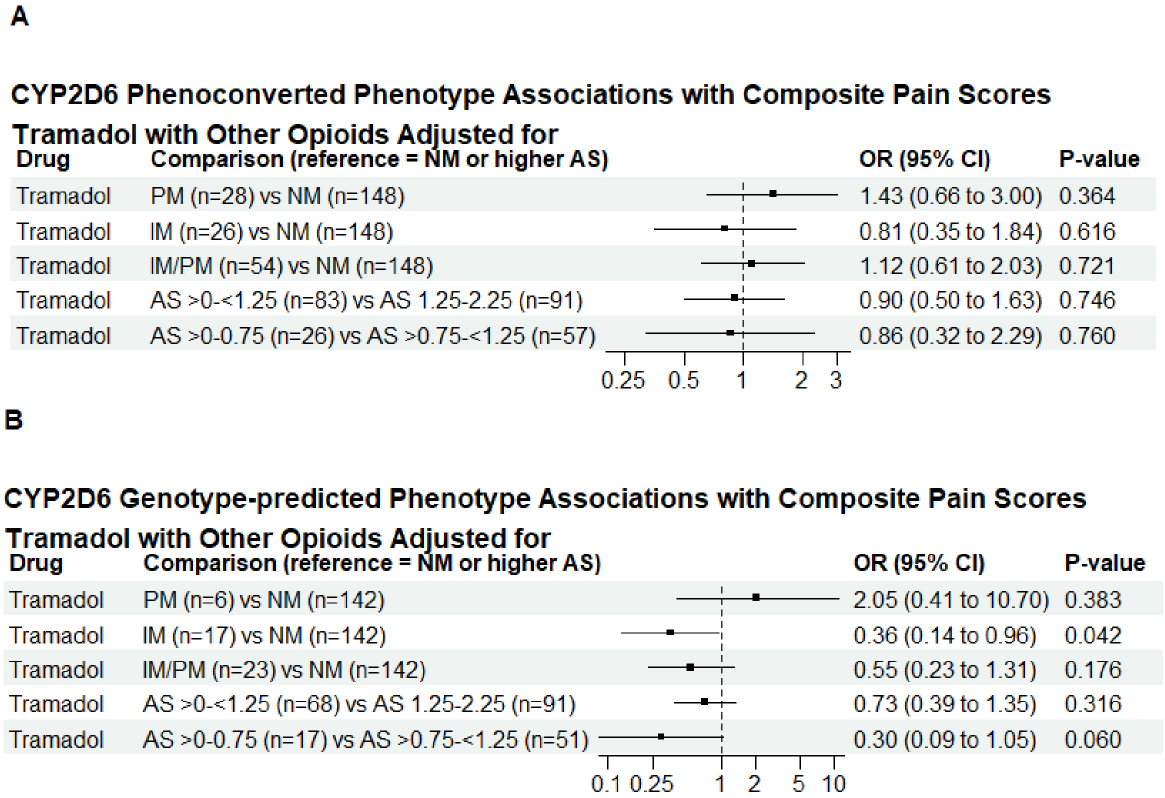

Supplementary Figure 2. CYP2D6 Phenoconverted and Genotype-predicted Phenotype Associations with Composite Pain Scores in Patients Taking Tramadol. Forest plots depict adjusted odds ratios (with 95% confidence intervals) for composite pain scores at 10 (±3) days after surgery across CYP2D6 phenotype groups taking tramadol, with concomitant use of other opioids included as covariates in all models. (A) Results based on phenoconverted (incorporating CYP2D6 inhibitor effect) CYP2D6 phenotypes; (B) Results based on genotype-predicted phenotypes (excluding patients on CYP2D6 inhibitors). NMs were used as the reference group for phenotype comparisons (PM vs NM, IM vs NM, and IM/PM vs NM). For AS subgroup comparisons, the higher AS group (AS 1.25-2.25 or AS >0.75-<1.25) was used as the reference. OR values <1 indicate lower pain scores relative to the reference group. ORs and CIs were estimated from ordinal logistic regression models, adjusting for age, race, sex, surgical procedure type, trial site group, acetaminophen, nonsteroidal anti-inflammatory drugs, gabapentinoids, serotonin and norepinephrine reuptake inhibitors, nerve block administration, and other opioid use. AS, activity score; CI, confidence interval; IM, intermediate metabolizer; NM, normal metabolizer; OR, odds ratio; PM, poor metabolizer.

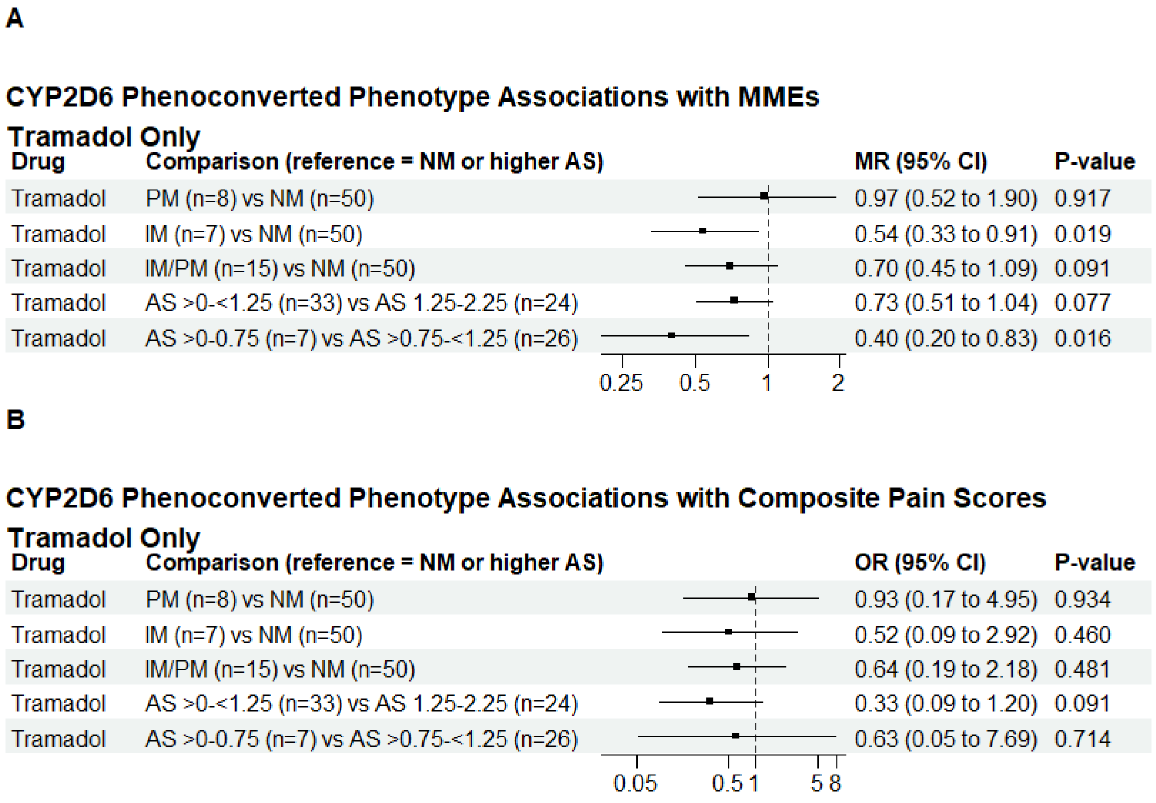

Supplementary Figure 3. CYP2D6 Phenoconverted Phenotype Associations with (A) Cumulative Tramadol Morphine Milligram Equivalents, and (B) Composite Pain Scores in Patients Taking Tramadol. Forest plots depict adjusted mean ratios (with 95% confidence intervals) for cumulative tramadol morphine milligram equivalents at 10 (±3) days after surgery and adjusted odds ratios (with 95% confidence intervals) for composite pain scores at 10 (±3) days after surgery across CYP2D6 phenotype groups taking tramadol only, without concomitant use of other opioids. NMs were used as the reference group for phenotype comparisons (PM vs NM, IM vs NM, and IM/PM vs NM). For AS subgroup comparisons, the higher AS group (AS 1.25-2.25 or AS >0.75-<1.25) was used as the reference. MR values <1 indicate lower opioid use relative to the reference group. OR values <1 indicate lower pain scores relative to the reference group. MRs and CIs were estimated from generalized linear regression models using a gamma distribution, whereas ORs and CIs were estimated from ordinal logistic regression models. All models were adjusted for age, race, sex, surgical procedure type, trial site group, acetaminophen, nonsteroidal anti-inflammatory drugs, gabapentinoids, serotonin and norepinephrine reuptake inhibitors, and nerve block administration. AS, activity score; CI, confidence interval; IM, intermediate metabolizer; MMEs, morphine milligram equivalents; MR, mean ratio; NM, normal metabolizer; OR, odds ratio; PM, poor metabolizer.
